# Small Molecule Antagonists of the Wnt/Beta-Catenin Signaling Pathway Target Breast Tumor-Initiating Cells in a Her2/Neu Mouse Model of Breast Cancer

**DOI:** 10.1371/journal.pone.0033976

**Published:** 2012-03-28

**Authors:** Robin M. Hallett, Maria K. Kondratyev, Andrew O. Giacomelli, Allison M. L. Nixon, Adele Girgis-Gabardo, Dora Ilieva, John A. Hassell

**Affiliations:** Department of Biochemistry and Biomedical Sciences, Centre for Functional Genomics, McMaster University, Hamilton, Ontario, Canada; The University of Hong Kong, Hong Kong

## Abstract

**Background:**

Recent evidence suggests that human breast cancer is sustained by a minor subpopulation of breast tumor-initiating cells (BTIC), which confer resistance to anticancer therapies and consequently must be eradicated to achieve durable breast cancer cure.

**Methods/Findings:**

To identify signaling pathways that might be targeted to eliminate BTIC, while sparing their normal stem and progenitor cell counterparts, we performed global gene expression profiling of BTIC- and mammary epithelial stem/progenitor cell- enriched cultures derived from mouse mammary tumors and mammary glands, respectively. Such analyses suggested a role for the Wnt/Beta-catenin signaling pathway in maintaining the viability and or sustaining the self-renewal of BTICs *in vitro*. To determine whether the Wnt/Beta-catenin pathway played a role in BTIC processes we employed a chemical genomics approach. We found that pharmacological inhibitors of Wnt/β-catenin signaling inhibited sphere- and colony-formation by primary breast tumor cells and primary mammary epithelial cells, as well as by tumorsphere- and mammosphere-derived cells. Serial assays of self-renewal *in vitro* revealed that the Wnt/Beta-catenin signaling inhibitor PKF118–310 irreversibly affected BTIC, whereas it functioned reversibly to suspend the self-renewal of mammary epithelial stem/progenitor cells. Incubation of primary tumor cells *in vitro* with PKF118–310 eliminated their capacity to subsequently seed tumor growth after transplant into syngeneic mice. Administration of PKF118–310 to tumor-bearing mice halted tumor growth *in vivo*. Moreover, viable tumor cells harvested from PKF118–310 treated mice were unable to seed the growth of secondary tumors after transplant.

**Conclusions:**

These studies demonstrate that inhibitors of Wnt/β-catenin signaling eradicated BTIC *in vitro* and *in vivo* and provide a compelling rationale for developing such antagonists for breast cancer therapy.

## Introduction

Recent findings suggest that human tumors exist as cellular hierarchies composed of tumorigenic and non-tumorigenic cells [1]. The occurrence of functionally-distinct tumorigenic cell compartments was demonstrated by separating tumor cells into different fractions based on their expression of cell surface markers, and transplanting the various fractions into immune-deficient mice [2]. Only some tumor cell fractions were capable of engrafting and eliciting tumor growth in mice, whereas others could not, even when large numbers of cells were transplanted suggesting that only a subset of tumor cells is capable of initiating tumor growth. Tumor-initiating cells (TICs), also commonly termed cancer stem cells, are thought to drive tumor growth, seed metastases and account for tumor relapse after remission [1]. In this model of tumor cell biology, TICs self-renew and differentiate giving rise to both tumorigenic and non-tumorigenic cells that make up the bulk neoplastic cell population. For example, human breast tumors can be fractionated into tumorigenic and non-tumorigenic cell populations based on their expression of the cell surface markers CD44 and CD24 [3]. Whereas only 0.01% of the bulk tumor cells were capable of seeding tumor growth in NOD/SCID mice, 0.5% of the CD44^+^CD24^−/low^: Lineage^−^ fraction was able to engraft and give rise to tumors. Furthermore, the tumor xenografts seeded by cells from the CD44^+^CD24^−/low^: Lineage^−^ fraction comprised the same cellular heterogeneity observed in the original tumor. TICs were originally identified in leukemia [2], and more recently in epithelial tumors of the breast [3], brain [4], prostate [5], gastro-intestinal tract [6], [7], skin [8], ovaries [9], and head and neck [10]. Hence TICs underlie a significant proportion of all malignancies.

The discovery of TICs has important implications for cancer therapy, namely that cancer treatments need to eliminate these cells to provide durable cure. Most current anticancer therapies were discovered based on their capacity to kill proliferating human tumor cell lines and to shrink xenografts in mice that were seeded by these same cell lines [11], [12]. However, tumor cell lines and their xenografts generally comprise relatively few TICs and as a result most current chemotherapies target the non-tumorigenic cells, which make up the bulk tumor mass. TICs possess increased chemotherapeutic resistance [13], [14], [15], [16], [17], [18], and decreased sensitivity to radiation therapy [19], [20], [21], properties that allow them to circumvent the killing effects of these commonly used anticancer agents. Hence TICs likely survive frontline cancer therapeutics and may account for cancer recurrence. The key to providing long-term cancer cure is to find a means to destroy TIC or abrogate their tumorigenicity thus eliminating tumor recurrence.

Identifying signaling pathways required for the survival and self-renewal of BTICs but not for their mammary epithelial stem cell counterparts may provide new molecular targets for anticancer drug discovery. However, studies of human BTICs have been confounded by their scarcity in tumors, the inability to isolate them as pure populations and means to readily culture them *in vitro* under conditions that maintain their tumorigenicity [2], [3], [4], [5], [6], [7], [10]. Additionally, most studies of human BTICs have not compared these cells to their normal stem cell counterparts. Such comparative analyses are likely necessary if we are to find therapies that selectively eradicate BTICs while sparing normal stem cells.

To overcome these limitations of human BTIC, we've investigated these cells in breast cancer-prone transgenic mice such as those that model ERBB2-positive breast cancer [22]. We found that mammary tumors of transgenic mice comprise ∼30% BTICs and that cells from these tumors can be propagated *in vitro* as non-adherent tumorspheres, which also comprise a similarly high fraction of BTICs [23]. We have also used these same culture conditions to propagate mouse mammary epithelial stem and progenitor cell cultures as non-adherent mammospheres, which serve as a normal stem/progenitor cell controls to compliment our studies of BTICs [23]. Here, we exploit the mouse breast cancer model to demonstrate that agents, which inhibit Wnt/Beta-catenin signaling, selectively target BTIC.

## Results

### Expression of Wnt/β-catenin signaling pathway components and target genes

**Figure 1:**
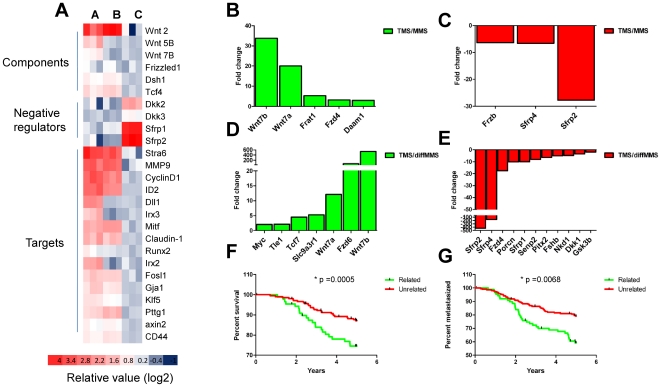
Expression of Wnt pathway genes in tumorspheres (TMS), mammospheres (MMS) and mammospheres induced to differentiate (diffMMS). A) Heat maps of 3 independent TMS (A, n = 3), MMS (B, n = 3) and diffMMS (C, n = 3) RNA preparations profiled on MOE430A Gene Chips. B–E) qRT-PCR of transcripts encoding components and target genes of the Wnt signaling pathway (* p<0.05, t-test) for all genes shown. F and G) Survival, (*p = 0.005, Log-rank test) and metastasis (*p = 0.0068, Log-rank test), curves for human breast cancer patients classified based on their expression of TMS-related and TMS-unrelated (diffMMS-related) specific Wnt pathway components.

Numerous previous studies have linked activation of Wnt/β-catenin signaling with breast cancer [21], [24], [25], [26], [27], [28], [29], [30], [31], [32], [33], [34]. We sought to extend these findings by first investigating the expression of Wnt/β-catenin signaling pathway components in mouse tumorspheres, mammospheres, and mammospheres induced to differentiate *in vitro*, which we used as approximate *in vitro* models of BTICs, mammary epithelial stem and progenitor cells, and differentiated mammary epithelial cells, respectively. A microarray analysis of 3 independent tumorsphere cultures established from independent mouse mammary tumors and 3 separate preparations of mammospheres and mammospheres induced to differentiate *in vitro*, revealed that the transcripts of many Wnt/β-catenin signaling pathway genes were most highly expressed in tumorspheres relative to either mammospheres or mammospheres induced to differentiate (Fig. 1A). The latter included upstream pathway components such as Wnt ligands, receptors, as well as the transcriptional co-activator TCF4 (Fig. 1A). Several Wnt/β-catenin target genes were also most highly expressed in tumorspheres, including Axin2, cyclinD1 and CD44. Interestingly, the expression of transcripts encoding inhibitory Wnt/β-catenin signaling pathway elements, such as Sfrp1, Srfp2 and Dkk2, were highest in mammospheres induced to differentiate, compared to both mammospheres and tumorspheres (Fig. 1A).

To verify the global gene expression profiling data we performed quantitative RT-PCR with primers that identified transcripts encoding components and downstream targets of the Wnt/β-catenin signaling pathway using additional independent preparations of RNA isolated from tumorspheres (n = 3), mammospheres (n = 3), and mammospheres induced to differentiate (n = 3), respectively. These analysis confirmed our original findings, namely that transcripts of components and target genes of the Wnt/β–catenin signaling pathway were generally more highly expressed in tumorspheres compared to either mammopsheres or mammopsheres induced to differentiate *in vitro* (Fig. 1B–E, *p<0.05 for all genes shown, t-test). For example, Wnt7A and 7B, as well as the Fzd4 and 6 receptors were most highly expressed in tumorspheres compared to either mammospheres or mammospheres induced to differentiate *in vitro* (Fig. 1 B&D). By contrast, mammospheres and mammospheres induced to differentiate expressed higher levels of transcripts that encode negative regulators of the Wnt pathway, such as Frzb, Sfrp2 and Sfrp4 (Fig. 1 C & E).

We also investigated whether the finding of differential expression of Wnt/β-catenin signaling pathway components and target genes using the *in vitro* models were relevant to human breast cancer patients. To this end we used whole tumor gene expression profiles derived from human breast tumor RNA samples with accompanying overall- and metastasis free survival data [35]. We identified a Wnt-based BTIC gene signature that comprised differentially expressed genes between tumorspheres and mammospheres that were induced to differentiate *in vitro.* We mapped these genes onto their human orthologs present in the previously published NKI whole tumor gene expression data set (Table 1). We then used the gene signature to learn whether we could separate patient tumor specimens from the NKI data set into tumorsphere-related and mammospheres induced to differentiate-related groups (tumorsphere-unrelated) [36]. Notably, the expression of tumorsphere-related Wnt/β-catenin signaling pathway genes was linked to poor overall survival (Fig. 1F, *p = 0.0005, log-rank test) and decreased metastasis-free survival (Fig. 1G, *p = 0.0068, log-rank test) when compared with the expression of mammospheres induced to differentiate-related Wnt/β-catenin signaling pathway genes. These data suggests that activated Wnt/β-catenin signaling may be a unique feature of BTICs derived from mouse mammary tumors relative to mammary epithelial stem and progenitor cells, and that Wnt/β-catenin pathway activation in BTICs may be linked to human breast cancer patient outcome and metastasis.

**Table 1:** Wnt/β-catenin pathway genes comprising the gene signature of differentially expressed genes between tumorspheres and mammospheres induced to differentiate.

Expressed in tumorspheres	Expressed in mammospheresinduced to differentiate
Wnt7A	Sfrp1
Myc	Sfrp4
Tle1	Fzd4
Tcf7	Senp2
SLC9A3R1	Pitx2
FZD6	Fshb
	Dkk1
	Gsk3β

Genes shown are those that were differentially expressed between tumorspheres and mammospheres induced to differentiate, as assessed by RT-PCR, and were also present in the NKI dataset.

### Wnt/β-catenin pathway agonists and antagonists regulate self-renewal and proliferation of BTIC and mammary epithelial stem/progenitor cells

**Figure 2:**
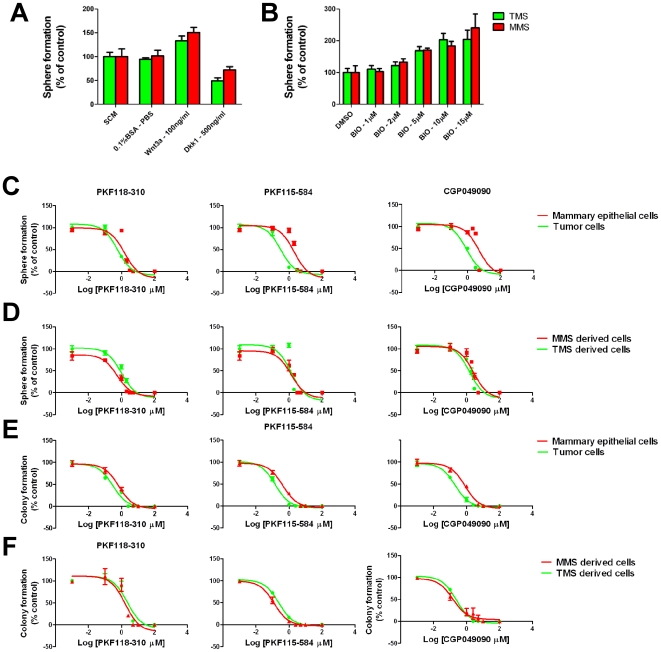
Agonists and antagonist of Wnt/β-catenin signaling regulate sphere and colony formation by primary tumor cells and primary mammary epithelial cells as well as by tumorsphere- and mammosphere-derived cells. A) Wnt3a and Dkk1 inhibit sphere formation by tumorsphere- and mammosphere-derived cells, compared to bovine serum albumin (BSA) and stem cell media (SCM) controls. B) BIO stimulates tumorsphere and mammosphere formation. C–D) Sphere formation in the presence of increasing concentrations of PKF118–310, PKF115–584, and CGP049090. E–F) Colony formation in the presence of increasing concentrations of PKF118–310, PKF115–584, and CGP049090.

To determine whether Wnt/β-catenin signaling was required for BTIC and mammary epithelial stem and progenitor cell function *in vitro*, we examined the effect of agonists and antagonists of Wnt/β-catenin signaling on sphere formation, which when performed under appropriate conditions [37] is thought to be an attribute of stem/progenitor cells and TICs [37], [38], [39], [40]. We seeded dispersed cells dissociated from mammospheres and tumorspheres into media containing either Dkk1 or Wnt3a at concentrations previously shown to be sufficient to inhibit or activate the Wnt/β-catenin signaling pathway, respectively [41], [42]. Dkk1 is a secreted protein inhibitor [43], and Wnt3A is a stimulatory ligand of Wnt/β-catenin signaling. Addition of Dkk1 reduced sphere formation, whereas addition of Wnt3A stimulated sphere formation of both mammosphere- and tumorsphere-derived cells (Fig. 2A). We used the small molecule BIO to inhibit GSKβ, a negative regulator of Wnt/β-catenin signaling [44]. Addition of BIO at various concentrations stimulated sphere formation by both mammospheres- and tumorsphere-derived cells in a dose-dependent manner (Fig. 2B).

We similarly tested pharmacological agents, which target Wnt/β-catenin signaling (PKF118–310, PKF115–584, CGP049090), for their affect on sphere formation. These inhibitors are specific antagonists of Wnt/β-catenin signaling that interrupt the penultimate step in pathway activation, namely the interaction between β-catenin and Tcf/Lef transcription factors [45]. We seeded freshly isolated primary tumor cells and primary mammary epithelial cells, as well as mammosphere- and tumorsphere-derived cells into medium containing various concentrations of the inhibitors. Each inhibitor reduced sphere formation by primary tumor cells and primary mammary epithelial cells (Fig. 2C), as well as by mammosphere- and tumorsphere-derived cells (Fig. 2D), in a dose dependent fashion. Notably, the inhibitory concentration of the compounds required to reduce sphere formation by 50% (IC_50_) (Table 2) did not significantly differ from those IC_50_ values reported previously to affect reduce Tcf-dependent reporter gene expression in cell lines (Table 2) [45].

**Table 2:** IC_50_ values (µM) for PKF118–310, PKF115–584, and CGP049090 in sphere forming assays.

Drug	Primary tumor Cells	Primary mammary epithelial cells	Tumorsphere-derived cells	Mammosphere-derived cells
**PKF118–310**	0.58	1.54	0.94	0.54
**PKF115–584**	0.31	2.05	1.34	1.39
**CGP049090**	0.84	4.89	1.52	2.64

IC_50_ calculations for the indicated Wnt/β-catenin pathway inhibitors assessed in sphere forming assays with primary tumor and mammary epithelial cells, as well as tumorsphere and mammosphere derived cells.

We also assessed the effects of the compounds on colony formation, an assay commonly used to enumerate stem and progenitor cells [46]. We seeded freshly isolated primary tumor cells and primary mammary epithelial cells, as well as mammosphere- and tumorsphere-derived cells at clonal cell density into collagen-coated plates in serum-containing medium. When colonies appeared, generally a week later, they were stained and counted. The inhibitors reduced colony formation by primary tumor cells and mammary epithelial cells (Fig. 2E), as well as that of mammosphere- and tumorsphere derived cells in a concentration dependent fashion (Fig. 2F). Inhibition of both colony and sphere formation occurred at similar inhibitor concentrations, suggesting that the inhibitors targeted both progenitor cells and stem cells as well as their tumor equivalent counterparts.

**Figure 3:**
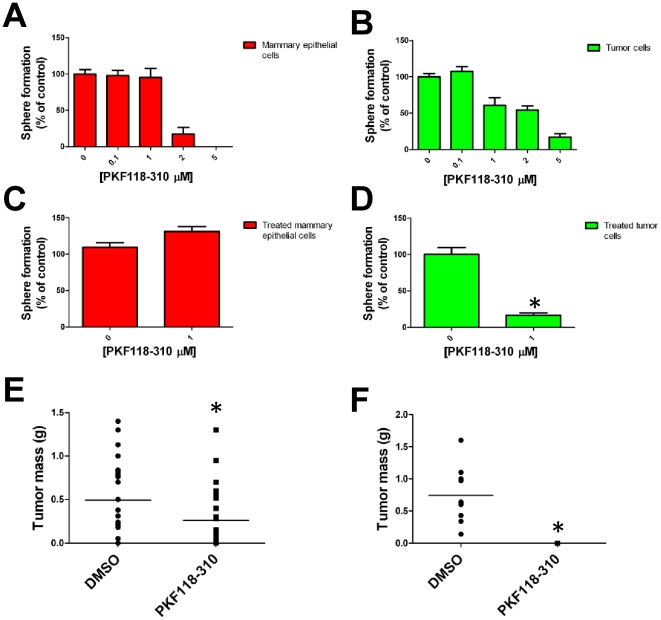
PKF118–310 selectively targets breast TICs *in* vitro. A) PKF118–310 inhibits sphere formation by primary mammary epithelial cells. B) PKF118–310 inhibits sphere formation by primary tumor cells. C) PKF118–310 treated primary mammary epithelial cells form spheres with same efficiency compared to the DMSO vehicle. D) PKF118–310 treated primary tumor cells have little capacity to form spheres compared to the DMSO vehicle (* p<0.05, t-test). E) Mass (g) of tumors formed from transplant of 1 µM PKF118–310-treated primary tumor cells (* p<0.05, t-test). F) Primary tumor cells treated with 2.5 µM PKF118–310 are unable to initiate tumor growth after transplant into syngeneic recipient mice.

To learn whether the inhibitory compounds affected the self-renewal of sphere-forming cells, we investigated the capacity of cells exposed to the compounds to serially form spheres. In short, dispersed primary mammary epithelial cells and primary tumor cells were exposed to PKF118–310 during a 4-day primary sphere-forming assay. The spheres that formed were counted, the cells dissociated from the spheres and the dispersed cells plated to form secondary spheres in fresh medium lacking the inhibitors. As we previously demonstrated, PKF118–310 reduced primary sphere-formation by both primary tumor cells and primary mammary epithelial cells dependent on its concentration (Fig. 3A&B). However, whereas the primary mammary epithelial cells treated with 1 µM PKF118–310 formed new mammospheres in secondary sphere-forming assays at the same frequency as the vehicle-treated mammary epithelial cells (Fig. 3C), the primary tumor cells exposed to 1 µM PKF118–310 exhibited a ∼10 fold reduced capacity to form spheres relative to the vehicle-treated primary tumor cells (Fig. 3D, *p<0.05). These observations demonstrate that a single exposure of tumor cells to PKF118–310 was sufficient to block their capacity to subsequently form spheres, even after PKF118–310 was no longer present in the medium. Conversely, mammary epithelial cells similarly exposed to PKF118–310 were not impaired in their sphere forming capacity, suggesting that the irreversible effect of PKF118–310 was limited to the primary tumor cells. These findings suggest that PKF118–310 reversibly affected the self-renewal of mammary epithelial cells *in vitro* but irreversibly affected the self-renewal of the primary breast tumor cells.

Our data suggesting that knock down of Wnt/β-catenin signaling by PKF118–310 irreversibly blocked the self-renewal of tumorsphere-initiating cells *in vitro,* prompted us to test whether this inhibitor specifically affected the capacity of BTICs to elicit tumor growth in syngeneic mice after tumor cell transplant. We incubated freshly isolated primary tumor cells under sphere forming conditions in presence of vehicle (DMSO) or PKF118–310 (1 µM or 2.5 µM concentrations). After 4 days the spheres were collected, dissociated and equal numbers (5,000) of viable PKF118–310- and vehicle-treated cells were injected sub-cutaneously into the syngeneic mice. Upon endpoint, when the tumor in any individual mouse reached 10% of its weight (∼6 weeks) all the animals were sacrificed and the tumors harvested. Tumors that arose from tumor cells incubated with PKF118–310 (1 µM) appeared with longer latency and were approximately 50% smaller at endpoint than those that arose from vehicle-treated tumor cells (control [m = 0.49 g], treatment [m = 0.26 g], *p<0.05) (Fig. 3E). Furthermore, when higher doses of PKF118–310 (2.5 µM) were used in these experiments, the tumor cells exposed to this concentration of the compound failed to initiate tumor growth in any of the host mice (Fig. 3F, *p<0.05, ∼6 weeks). Collectively, these data demonstrate that incubation of primary tumor cells with PKF118–310 for 4 days *in vitro* substantially reduced BTIC frequency.

### PKF118–310 halts tumor growth *in vivo*

**Figure 4:**
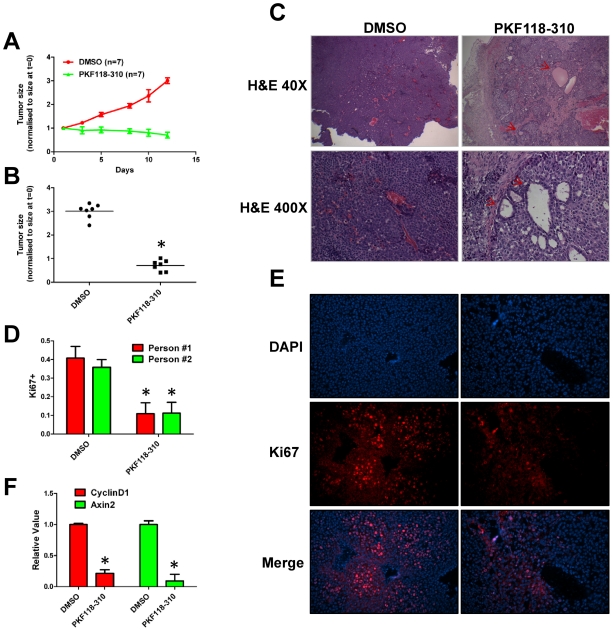
PKF118–310 treatment halts tumor growth. A) Tumor volumes of vehicle and PKF118–310 treated mice. B) Tumor volumes after completion of treatment (t = 12 days) (*p<0.05, t-test). C) PKF118–310 induces formation of duct-like structures (red arrows). D) Photographs of Ki67 stained tumor sections. E) PKF118–310 reduces the fraction of Ki67+ nuclei; quantification of Ki67-positive nuclei was assessed independently by two different individuals (*p<0.05, t-test). F) Quantification of Wnt target gene expression in tumors isolated from treated and untreated mice using qRT-PCR (*p<0.05, t-test).

Our data suggested that incubation of primary breast tumor cells *in vitro* with PKF118–310 substantially reduced BTIC frequency as assessed by cell transplant into syngeneic mice. To determine whether PKF118–310 affected the growth of pre-established breast tumors and reduced BTIC frequency *in vivo*, we transplanted primary breast tumor cells into syngeneic mice and treated these hosts with PKF118–310 after the tumors had reached a volume of ∼1 cm^3^. We found that treating tumor-bearing mice with 0.85 mg/kg of PKF118–310 for 12 days (5 days on, 2 days off and 5 days on) inhibited tumor growth compared to their vehicle-treated counterparts, but did not induce significant tumor regression during the treatment period (Fig. 4A). After treatment ceased the tumors in mice administered the vehicle were ∼3 fold larger than those in PKF118–310-treated mice (Fig. 4B, *p<0.05).

To uncover potential mechanisms whereby the compound halted tumor growth, we prepared sections from the tumors of both cohorts and stained them with Hematoxylin and Eosin (H&E). Interestingly the tumors of the mice treated with PKF118–310 contained many cell-free areas that were often circumscribed by a ring of cells resembling a duct (Fig. 4C). In some cases the cell-free areas contained material, characteristic of milk protein secretions. Interestingly, the changes in histology induced by treatment with PKF118–310 are consistent with reduced tumor grade, a histological parameter associated with better survival outcomes in human breast cancer patients [47]. We also examined whether inhibition of tumor growth was accomplished by a reduction in cell proliferation and/or induction of apoptosis. We stained tumor sections for markers of proliferation (Ki67) and apoptosis (cleaved caspase-3 and TUNEL). We observed a significant decrease in the frequency of Ki67 positive tumor cell nuclei in the PKF118–310 treated tumor-bearing mice compared to their vehicle-treated counterparts (Fig. 4D&E). We did not observe any positive staining for cleaved caspase-3 or TUNEL in tumors from either vehicle- or PKF118–310-treated mice (data not shown). Tumors from PKF118–310 treated mice comprised approximately 3–4-fold fewer Ki67-positive cells than tumors from vehicle-treated mice. We similarly simultaneously stained the tumor sections with antibodies to a luminal lineage marker (CK8) and with those to each of two myoepithelial lineage markers (CK14 and alpha-smooth muscle actin [alpha-SMA). The vast majority of the cells in sections prepared from tumors of the vehicle-treated mice expressed only the luminal lineage marker in keeping with previous findings (data not shown). Tumor sections prepared from tumors of the mice administered PKF118–310 also only expressed the luminal lineage marker. Surprisingly the cells comprising the duct-like structures found in tumor sections of mice administered PKF118–310 expressed the luminal lineage marker but not either of the myoepithelial lineage markers.

To learn whether administration of PKF118–310 to tumor-bearing mice inhibited Wnt/β-catenin signaling in tumors, we measured the abundance of Wnt/β-catenin target gene transcripts in the tumor cells. The abundance of both axin2 and cyclin D1 transcripts was significantly lower in tumors harvested from PKF118–310-treated mice compared to their vehicle-treated counterparts, confirming that PKF118–310 targeted Wnt/β-catenin signaling *in vivo* (Fig. 4F).

**Figure 5:**
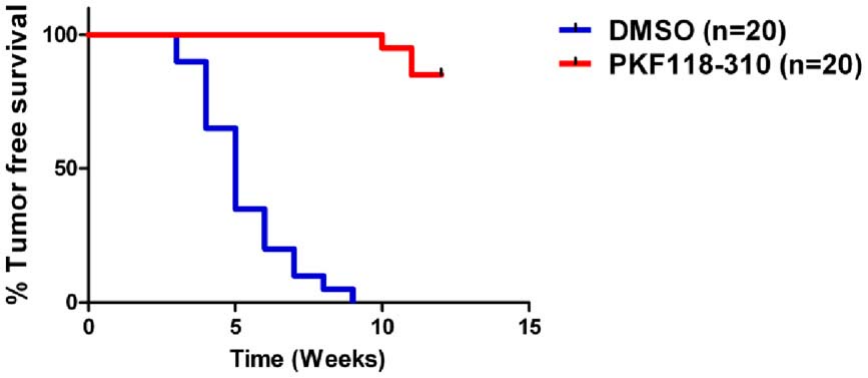
Tumor cells from PKF118–310 treated tumor-bearing mice engraft and elicit the growth of secondary tumors less efficiently than their vehicle-treated counterparts. Viable tumor cells were harvested from PKF118–310- and DMSO-treated mice and 10,000 viable tumor cells per mouse were transplanted into syngeneic recipients (n = 20 treated/untreated) (* p<0.05, Log-rank test).

The principal objective of our experiments was to learn whether inhibition of Wnt/β-catenin signaling targeted BTICs in tumors. Because, treatment with PKF118–310 did not completely shrink tumors *in vivo,* we wondered whether PKF118–310 eradicated functional BTICs in the tumors of treated mice. We reasoned that if PKF118–310 selectively targeted BTICs *in vivo,* viable cells isolated from PKF118–310 treated tumor-bearng hosts would engraft and elicit tumor growth less efficiently relative to their vehicle-treated counterparts. To this end, we transplanted tumor cells by injecting them sub-cutaneously (n = 20, PKF118–310-treated; n = 20, vehicle-treated) between the shoulders of syngeneic mice (10,000 cells/mouse) and measured tumor latency in the PKF118–310-treated and vehicle-treated transplant cohorts. Mice transplanted with tumor cells harvested from vehicle-treated mice experienced a median of 5-week tumor free survival and all mice had palpable tumors after 9 weeks. By contrast, tumor cells harvested from PKF118–310 treated mice generally failed to initiate tumor growth. In fact, 85% of mice transplanted with PKF118–310 treated tumor cells remained tumor free over a 12 week follow-up period (Fig. 5). The tumors arising following cell transplant were invariably lodged in the #2 mammary fat pad, which extends from the ventral to the dorsal area of the host mice (data not shown).

Together with our previous observations, these results demonstrate that not only was PKF118–310 treatment sufficient to halt tumor-growth *in vivo,* but that cells comprising the tumors of mice administered the compound were substantially diminished in their capacity to engraft and initiate tumor growth compared to their vehicle-treated counterparts.

## Discussion

Whereas TIC have been identified in a wide variety of human and mouse malignancies [2], [3], [4], [5], [6], [8], [48], [49], [50], little is known about their underlying biology, and few compounds have been identified that selectively target these cells [51], [52]. Targeting TICs is an important cancer therapeutic objective as these cells are resistant to current cancer therapies, including chemo- and radiation-therapy [14], [17], [19], [20]. Hence whereas standard therapies result in tumor shrinkage, they may fail to provide long lasting cures because rare TIC survive and seed tumor relapse.

The use of genomic and drug discovery technologies, such as global gene expression profiling and high-throughput screening would greatly aid the search for anti TIC therapies. However, implementation of these methodologies has been confounded by a lack of suitable human BTIC-enriched populations for study. Typically BTIC represent an exceedingly small percentage (∼0.01%) of the total tumor cell population [3], [53], and even in the most highly enriched populations, BTIC rarely achieve more than 1–2% purity [3], [53]. Moreover, means of propagating BTIC-enriched tumor cell populations *in vitro* have not been described. To overcome these obstacles, we have studied BTICs from mouse mammary tumors of breast cancer prone transgenic models because they comprise a high BTIC frequency, averaging ∼30% in most tumors and companion tumorspheres [23].

To determine whether the Wnt/Beta-catenin pathway is required for the survival and/or self-renewal of BTIC, we employed three small-molecular weight tool compounds, PKF118–310, PKF115–584 and CGP049090, which were originally identified in a high throughput screen to identify those that abrogate the binding of β-catenin to Tcf4 *in vitro* [45]. Follow up analyses of these compounds revealed their capacity to: block β-catenin binding to GST-Tcf4 *in vivo*; reduce expression of a Wnt/β-catenin luciferase reporter; restore the β-catenin induced axis duplication of *Xenopus* embryos when co-injected with β-catenin; inhibit expression of the Wnt target genes *Myc* and *CyclinD1*; and retard the proliferation of colon cancer cell lines known to display hyperactive Wnt signaling *in vitro* [45]. Collectively, the latter findings suggest that PKF118–310, PKF115–584 and CGP049090 reduce Wnt/β-catenin signaling leading to the inhibition of cancer cell line proliferation *in vitro*. To the best of our knowledge the effect of these compounds on breast tumorigenesis has not previously been assessed.

Several studies have implicated Wnt/β-catenin signaling in both the pathogenesis of breast cancer and the regulation of normal mammary epithelial stem cell processes [24], [31], [54]. Our data suggests that the Wnt/β-catenin pathway is hyperactive in BTIC compared to normal mammary epithelial stem/progenitor cells or to their more differentiated descendants. We made use of the small molecule inhibitors to investigate the consequences of inhibiting Wnt/β-catenin signaling in both breast tumor cells and normal mammary epithelial stem/progenitor cell populations. Due to the limited availability of the natural compounds PKF115–584 and CGP049090, we focused primarily on the use of PKF118–310, which can be chemically synthesized. Our initial experiments showed that each of the 3 compounds inhibited sphere and colony formation by primary tumor cells and primary mammary epithelial cells, as well as by established tumorsphere- and mammosphere-derived cells without any apparent selectivity. However, both PKF115–584 and CGP049090 displayed somewhat increased selectivity of between 6–7 fold (IC_50_^MMS^/IC_50_^TMS^, Table 1) for primary tumor cells over primary mammary epithelial cells in sphere forming assays compared to PKF118–310 (2–3 fold selectivity), indicating that further investigation of the potential selectivity these compounds is warranted.

We did not observe any significant selectivity of PKF118–310 for either the survival and/or self-renewal of tumorsphere-initiating cells compared to mammosphere-initiating cells in primary sphere-forming assays (Fig. 2C–F). However, a single exposure of primary tumor cells to PKF118–310 in a primary sphere-forming assay was sufficient to block subsequent secondary sphere formation in the absence of the compound. By contrast, mammary epithelial cells exposed to PKF118–310 were not impaired in their capacity to form secondary spheres, suggesting that the effect of PKF118–310 on secondary sphere formation is specific to BTICs. Taken at face value these observations suggest that PKF118–310 inhibited tumorsphere formation by an irreversible mechanism, whereas the compound acted reversibly to affect mammosphere formation. Inhibition of Wnt/Beta-catenin signaling by PKF118–310 may be cytotoxic for tumorsphere-initiating cells, perhaps because they are addicted this pathway, whereas pathway inhibition may be cytostatic for mammosphere-initiating cells.

Whereas sphere formation is a convenient and relatively rapid surrogate *in vitro* assay for stem/progenitor and TIC activity, the nature of sphere-forming cells is controversial and consequently we employed additional means to identity the tumor cells that might be targeted by PKF118–310 [37], [38], [39], [40], [55]. To this end we transplanted primary tumor cell populations that had been incubated with PKF118–310 under the same conditions as had been used in primary sphere-forming assays and thereafter measured the capacity of the remaining viable tumor cells to seed tumor growth after transplantation into syngeneic FVB/N female mice. These transplantation assays directly assess BTIC frequency and demonstrated that PKF118–310 targeted these cells as manifested by a concentration-dependent reduction in tumor incidence in recipient mice resulting from transplant of the compound-treated tumor cells.

Administration of PKF118–310 to tumor-bearing mice blocked tumor growth during the 10-day treatment period, an interval during which the tumors expanded by 2–3 fold in tumor-bearing mice that were administered the vehicle. Histological analyses of tumor sections from mice administered PKF118–310 revealed loss of tumor architecture manifested as reduced cellularity and phenotypic features associated with reduced tumor grade. Whereas no evidence of apoptotic cell death or altered expression of differentiation markers was evident in tumor sections from mice administered the compound (data not shown), the frequency of Ki67-positive cells, a biomarker of cell proliferation, was markedly reduced. Importantly, tumor cells harvested from mice exposed to PKF118–300 formed tumor grafts in only 3 of 20 mice transplanted with these cells some 2 weeks after tumors had already formed in 20/20 mice transplanted with vehicle-treated tumor cells. Our transplantation assay can detect single tumor cells in the bulk tumor cell population, which on average comprise ∼30% functional BTIC as established by limiting dilution cell transplantation assays [23]. Hence, our findings suggest that the frequency of BTIC comprising the tumors was dramatically reduced by inhibiting Wnt/Beta-catenin signaling in tumors [23]. Taken together these multiple lines of investigation suggest that antagonists of Wnt/Beta-catenin signaling target BTIC and provide proof-of-principle that eradicating these cells leads to durable breast cancer remission.

## Materials and Methods

### Care and treatment of animals

All mice used in these experiments were housed in a Canadian Council on Animal Care (CCAC)-approved facility at McMaster University. Mice were provided with food and water *ad libitum*. All animal experiments were conducted in accordance with the requirements of the CCAC.

### Tumor and mammary epithelial cell culture

The #3 and #4 mammary glands from virgin female FVB/N mice (6–8 weeks old) and mammary tumors were isolated as described previously [23], [56]. Mammospheres and tumorspheres were established from the bulk primary mammary epithelial and tumor cell population respectively as described previously [57]. Serial passage of the mammospheres and tumorspheres was accomplished by mechanically dissociated the cells from spheres using titruation and reseeding the dispersed cells into fresh medium. Passage of the spheres was limited to 3–5 serial passages before the cells were harvested and RNA prepared. To induce a differentiation program in mammospheres *in vitro,* intact mammospheres were collected by centrifugation, the spheres were dissociated and the dispersed cells were plated at a density of 150,000 cells/ml on rat-tail collagen (Roche, Basel, Switzerland) coated 60 mm Petri dishes [58], [59]. The cells were incubated for a week before they were harvested and used to prepare cellular RNA for analyses.

### RNA isolation and analyses

Total RNA was isolated from tumorspheres, mammospheres or mammospheres induced to differentiate using an RNAeasy mini prep kit (QIAGEN, Hilden, Germany) according to the manufacturer's protocol. RNA was quantified using spectrophotometic analyses (A_260 nm_/A_280 nm_) and it's quality assessed by gel electrophoresis. RNA from 3 independent tumorsphere, mammosphere, and mammospheres induced to differentiate cell populations was used to prepare cRNA probes for hybridization to MOE430A Gene Chips™ in accordance with manufacturer's protocols (Affymetrix, Santa Clara, California). Gene expression profiling data was analyzed using Genespring™. Gene expression values were normalized to the average expression of either mammospheres induced to differentiate or tumorspheres for each probe set to generate a heat map. When a gene was represented by multiple probe sets, the most highly differentially expressed probe set was chosen for display in the heat map. These data have been deposited in the gene expression omnibus (GEO, GSE32463). Independent RNA preparations from different populations of tumorspheres, mammospheres, or mammospheres induced to differentiate was also analyzed by quantitative RT-PCR using the mouse Wnt Signaling Pathway RT^2^ *Profiler™* (QIAGEN). Total cellular RNA was isolated using the RNAeasy mini prep kit (QIAGEN) and used as template for oligo-dT primed reverse transcription using SuperScriptII™ First Strand Synthesis (Invitrogen, Carlsbad, California) for quantitative RT-PCR The abundance of selected mRNA transcripts were determined (primer sequences available upon request) with quantitative RT-PCR using FastStart DNA Master SYBR Green I Kit on the Light Cycler (Roche) according to the manufacturer's protocol.

### Gene signature

Microarray and clinical data was downloaded from http://microarray-pubs.stanford.edu/wound_NKI/explore.html. The expression of Wnt/β-catenin signaling pathway genes was used to divide patients into related and unrelated Wnt/β-catenin signature groups as previously described [36].

### Sphere, colony, and *ex vivo* treatment assays

Sphere and colony forming assays were completed as previously described [23]. Dkk1 and Wnt3A were obtained from RnDSystems (Minneapolis, Minnesota). PKF118–310, PKF115–584 and CGP049090 were a gift from Novartis (Basel, Switzerland).

### IC_50_ calculations

The 50% inhibitory concentration (IC_50_) of compounds was calculated using GraphPad Prism5 software. X-axis values were X = Log(X) transformed and then fit with a dose-response curve. The DMSO vehicle control was included to aid IC_50_ calculation and was assigned a 1 nM concentration of the tested compound.

### *In vivo* compound administration

Freshly isolated primary tumor (100,000) cells were suspended in 50% Matrigel (BD, Franklin Lakes, New Jersey), 45% phosphate buffered saline pH7.4 (PBS) and 5% fetal bovine serum (FBS) [Invitrogen, Carlsbad, California], and the cells were injected subcutaneously between the shoulders into syngeneic 6–8 week-old female mice (FVB/N strain). Mice were monitored by palpation weekly for the occurrence of tumors. When tumors reached roughly 1 cm^3^, the mice were administered either the vehicle (0.1% DMSO) or PKF118–310 (0.85 mg/kg) dissolved in 0.1% DMSO by intra-tumoral injection for 5 consecutive days followed by a 2-day rest period before compound administration was repeated once. Tumor volume was measured twice weekly. At the end of the 12-day treatment cycle the mice were sacrificed and their tumors harvested for analysis. We found that tumors were invariably embedded in the fat pads of the number 2 mammary glands. Viable tumor cells (assessed by TrypanBlue staining) from vehicle- and compound-treated mice were isolated as described above and 10,000 cells were transplanted subcutaneously into syngeneic mice (n = 20, treated; n = 20, untreated).

### Histology and immuno-histochemical analysis

Paraformaldehyde fixed tumor fragments were embedded in paraffin, sectioned and stained with H&E. The tumor sections were de-paraffinized and rehydrated in ethanol (100-70% gradient) before immunofluorescent analysis. Antigen retrieval was performed in Antigen Unmasking Solution (Vector, Burlingame, California). Slides were blocked with 3% normal goat serum (Dako, Denmark) and incubated with primary antibodies for 2 hours at room temperature (Ki67, 1∶200 [ABCAM, Cambridge, Massachusetts]. Secondary antibodies (Invitrogen) were used at a 1∶200 dilution for 1 hour at room temperature.
